# Cognitive conflict and restructuring: The neural basis of two core components of insight

**DOI:** 10.3934/Neuroscience.2019.2.60

**Published:** 2019-05-21

**Authors:** Amory H. Danek, Virginia L. Flanagin

**Affiliations:** 1 Experimental and Theoretical Psychology, Universität Heidelberg, Heidelberg, Germany; 2 German Center for Vertigo and Dizziness (DSGZ), Klinikum der Universität München, Marchioninistr. 15, 81377 Munich, Germany

**Keywords:** insight, problem solving, restructuring, cognitive conflict, fMRI

## Abstract

Sometimes, the solution to a difficult problem simply pops into mind. Such a moment of sudden comprehension is known as “insight”. This fundamental cognitive process is crucial for problem solving, creativity and innovation, yet its true nature remains elusive, despite one century of psychological research. Typically, insight is investigated by using spatial puzzles or verbal riddles. Broadening the traditional approach, we propose to tackle this question by presenting magic tricks to participants and asking them to find out the secret method used by the magician. Combining this approach with cueing in an fMRI experiment, we were able to break down the insight process into two underlying components: cognitive conflict and restructuring. During cognitive conflict, problem solvers identify incongruent information that does not match their current mental representation. In a second step this information is restructured, thereby allowing them to correctly determine how the magic trick was done. We manipulated the occurrence of cognitive conflict by presenting two types of cues that lead participants to either maintain their perceptual belief (congruent cue) or to change their perceptual belief (incongruent cue) for the mechanism behind the magic trick. We found that partially overlapping but distinct networks of brain activity were recruited for cognitive conflict and restructuring. Posterior, predominantly visual brain activity during cognitive conflict reflected processes related to prediction error, attention to the relevant cue-specific sensory domain, and the default brain state. Restructuring on the other hand, showed a highly distributed pattern of brain activity in regions of the default mode, executive control networks, and salience networks. The angular gyrus and middle temporal gyrus were active in both cognitive conflict and restructuring, suggesting that these regions are important throughout the insight problem solving process. We believe this type of approach towards understanding insight will give lead to a better understanding of this complex process and the specific role that different brain regions play in creative thought.

## 1. Introduction

Moments of sudden enlightenment are known as “insight” or “Aha! moments”. Typically, after struggling with a difficult problem for a long time, the solution pops into mind suddenly and effortlessly, offering a completely new perspective on the seemingly unsolvable problem. Creative insight is an essential facet of human thinking and can be regarded as a ubiquitous process which is highly relevant for the scientific, technological or cultural advancement of society. Unfortunately, a feature of insight is that it occurs rarely, making it a difficult process to study systematically.

Insight can be defined as a “complex, non-linear transition process that consists of an affective component (the subjective Aha! experience) and a cognitive component (the sudden representational change or restructuring leading to a correct solution)” [1]. While it has been shown that the subjective Aha! experience is not always present during the solution process [2], the cognitive component (i.e. restructuring the mental problem representation from an initially incorrect one to a correct one) can be considered essential for solving the problem. Thus, we focused on the cognitive component of insight, without assessing the affective Aha! experience. An additional reason for this decision was that cues have been shown to alter solvers' subjective experience of the solution process [3],[4], letting Aha! ratings appear as a less useful measure in the present study which used cues as main manipulation. The aim of the present study was to break down the cognitive component of insight into two underlying cognitive processes thought to be crucially involved in insight problem solving and to look for neural activity specific to each of them: Cognitive conflict and restructuring.

“Cognitive conflict” in insight problem solving must be differentiated from response conflict where a decision has to be made between several competing actions, as for example in the Stroop task [5]. Rather, while working on a problem, solvers may detect incongruent information which does not fit with the current mental representation of the problem. This realization is likely to produce cognitive conflict. In some cases, this conflict cannot be resolved, leading problem solvers into an impasse. To resolve this conflict, the initial view on the problem must be modified which opens up new solution possibilities. Thus, detecting and processing cognitive conflict is likely to be one of the key processes involved in insight. In other words, cognitive conflict is needed in order to trigger a change in the problem representation, as postulated by Luo and Knoblich [6].

Conflict detection is a prerequisite of restructuring, which can be defined as “a change in the problem solver's mental representation of the problem” [7] or simply as “structuring again” [8]. The Gestalt psychologists were the first who described this cognitive process as a fundamental change in thinking which drastically alters the solver's view on a given problem—“Umzentrieren”, i.e. “re-centering”, as Max Wertheimer put it [9]. There is wide-spread agreement that restructuring is crucial for insight, for example Luo and Knoblich stated that “The process of restructuring is regarded as the essential feature of insight problem solving” [6]. Restructuring is the key process in the representational change theory of insight [7],[10]–[12] which has given the Gestalt tradition a modern, more precise and testable form. Ohlsson [11] has suggested different mechanisms how a problem representation can be restructured, for example by relaxing unnecessary constraints, directing attention towards the relevant problem features, recombining information or perceptual re-groupings of problem elements. Restructuring implies that the initial way of thinking about a problem must be overcome (since it does not lead to the correct solution) and be changed by new combinations of the given information. If a problem that initially triggers an incorrect representation is eventually solved correctly, we can infer that restructuring has taken place.

Only few studies exist so far that have tried to identify neural correlates of restructuring. An EEG study by Sandkühler and Bhattacharya [13] used self-reported restructuring and found right prefrontal activity. However, it is doubtful whether participants are able to consciously report on their experience of restructuring which is thought to mainly rely on unconscious processing [12]. Indeed, there is evidence that insight problem solving proceeds in an all-or-none fashion, with no partial solution information being available to the solver before the full solution is reached [14]. In the same vein, Metcalfe [15] found that solvers were not even able to predict their eventual success on insight problems nor were they able to correctly report their progress via feeling-of-warmth ratings [16].

It is for that reason that we chose, in the present study, not to operationalize restructuring through self-reports, but to rely instead on the more objective measure of solution rates, or to compare correct with incorrect solutions: solved with unsolved trials. By using a task which inherently triggers an initially incorrect problem representation that can only be solved if restructured, the occurrence of restructuring is inferred from whether the problem was correctly solved or not. If the problem is not solved (or not solved correctly), no restructuring has taken place. If the problem is solved correctly, restructuring has taken place. This design is similar to the Sandkühler study where she used a comparable contrast that she called “Deeper understanding” [13] as well as to another recent study by Tik et al. [17].

In addition to restructuring, this study also examined cognitive conflict using the common approach of giving cues to manipulate participants' mental representations [4]. Conflict was manipulated by presenting either incongruent cues that provide conflicting information, or congruent cues that produce no conflict (compare Methods). Congruent cues were consistent with participants' initial, faulty problem representation whereas incongruent cues contained new information that contradicted the initial way of thinking about the problem. Only the incongruent cues produce a cognitive conflict by forcing participants to question their initial problem representation. This conflict can be resolved by initiating a restructuring which may then enable solvers to find a solution.

The studies that have so far investigated the neural basis of insight have led to highly diverse results. The first studies focused on verbal tasks and already multiple brain regions were found, among them the right anterior superior temporal gyrus (STG) [18], left supramarginal gyrus and the anterior cingulate cortex, ACC [19], superior occipital gyrus, temporal gyrus, angular gyrus, precuneus, many frontal areas and the ACC [20] and the left lateral PFC as well as the ACC [21]. Reviewing the existing fMRI data, Dietrich and Kanso [22] concluded that while the ACC was consistently found and the STG was reliably activated at least by one type of verbal problems, these findings remained very heterogeneous and no clear picture emerged with regard to a more general basis of insight. Additionally, a right-hemispheric dominance had been postulated for insight [23],[18],[24], Dietrich and Kanso [22] however concluded that there is no support for this claim, neither in the reviewed electrophysiological data nor in the neuroimaging data.

Because all of the aforementioned studies implemented tasks from only one domain (verbal), these findings might be partially task-specific. A more recent quantitative meta-analysis on insight by Sprugnoli et al. [25] compared results across different task domains. They came to a similar conclusion as Dietrich and Kanso [22], that there was no evidence for right-brain dominance, and that the brain regions recruited for insight were highly diverse. According to this review, the following regions were all involved in insight problem solving: Precentral gyrus, middle temporal gyrus, precuneus, cingulate gyrus, claustrum, middle occipital gyrus, uvula (inferior vermis—cerebellum) and insula (all left hemisphere) and superior frontal gyrus, insula, precuneus and middle temporal gyrus (right hemisphere).

One reason for the lack of convergence across studies may be the widely differing designs and contrasts used. For example, in the Sprugnoli meta-analysis, the operationalization of “insight” ranged from giving cues [21] or showing solutions [26] to choosing between different solution alternatives [27] or comparing self-reported “Aha! solutions” with “no Aha! solutions” [28],[18]. Obviously, it is highly questionable whether these studies assessed insight in a comparable manner and thus the inconsistency is not very surprising. A perhaps more promising approach could be to tackle the individual components of this complex thinking process separately before trying to understand the bigger picture of insight.

The present study was designed with the aim of locating neural correlates of two individual components, cognitive conflict and restructuring, of the insight network. We tested whether neural activity would vary as a function of cue type to examine cognitive conflict. In addition, we aimed at identifying brain areas active during restructuring. Given the fact that there is no clear evidence for hemispheric dominance in insight problem solving [22],[25], we did not set up any specific hypotheses regarding laterality.

The present study implemented a set of magic tricks as a problem-solving task, asking participants to find out the secret method used by the magician. We have previously shown that magic tricks are well suited to investigate insight problem solving [1],[29] and have used them previously in a passive viewing paradigm to examine the neural correlates of expectation violation [30]. To gain insight into a magic trick requires a representational change, typically a change of conceptual knowledge about objects, e.g. realizing that a seemingly solid ball is only a half ball. The initial mental representation of the problem which is typically incorrect must be restructured into the correct problem representation which then allows to solve the problem. Following this rationale, we argue that magic tricks represent ideal material to investigate insight and specifically, the restructuring process, as also outlined by Danek [1]. In order to be able to trigger cognitive conflict and, subsequently, restructuring, a cueing paradigm was used, as described above. Behaviorally, we expected higher solving rates after incongruent cues, as compared to congruent ones. With regard to neural activity, we expected that the incongruent, but not the congruent cue will lead to cognitive conflict. Thus, neural correlates of cognitive conflict in insight problem solving will be identified through the following contrast: Incongruent cue (cognitive conflict) > congruent cue (no cognitive conflict). Neural correlates of restructuring in insight problem solving will be identified through the following contrast: Correct solutions (restructuring) > incorrect solutions (no restructuring).

## 2. Materials and method

### 2.1. Participants

Thirty-two healthy right-handed adults (mean age: 23.2 yrs, range 19–30 yrs; 16 male) participated in this experiment. Participants were only recruited if they had no contraindication for entering the MRI room (non-removable metal) and no history of neurological disease. All participants gave written informed consent to participate in the study, according to the Declaration of Helsinki, and were monetarily compensated with 20.-Euro for their time. The study was approved by the ethics committee of the medical faculty of the Ludwig-Maximilians-Universität München (#109-10). Two participants were excluded from the analysis because they stated in the solution phase (see Figure 3 and Procedure) that they had known the solution already before the cue was shown for 17 and 16 out of 20 tricks, respectively. In these participants, the number of trials in which cognitive conflict and restructuring could have occurred were too low for the analysis. The remaining 30 participants (mean age: 23.3 yrs, range 19–30 yrs; 15 male) were used in the analysis.

### 2.2. Testing material

#### 2.2.1. Magic tricks

A set of 20 magic tricks was presented to participants as a problem-solving task (“Please try to find out how the trick works!”). The material consisted of short video clips of magic tricks that were performed by a professional magician and recorded in a standardized setting. Clips ranged from 6.3 to 42.5 s. The magic stimuli covered a wide range of different magic effects (e.g. transposition, levitation, vanish) and techniques (e.g. misdirection, gimmicks, optical illusions). This paradigm has been established as a relatively new domain for insight problem solving [31]. All magic tricks used are difficult problems with low solving rates, with the characteristic that the initial, seemingly obvious problem representation does not lead to a correct solution.

**Figure 1.**
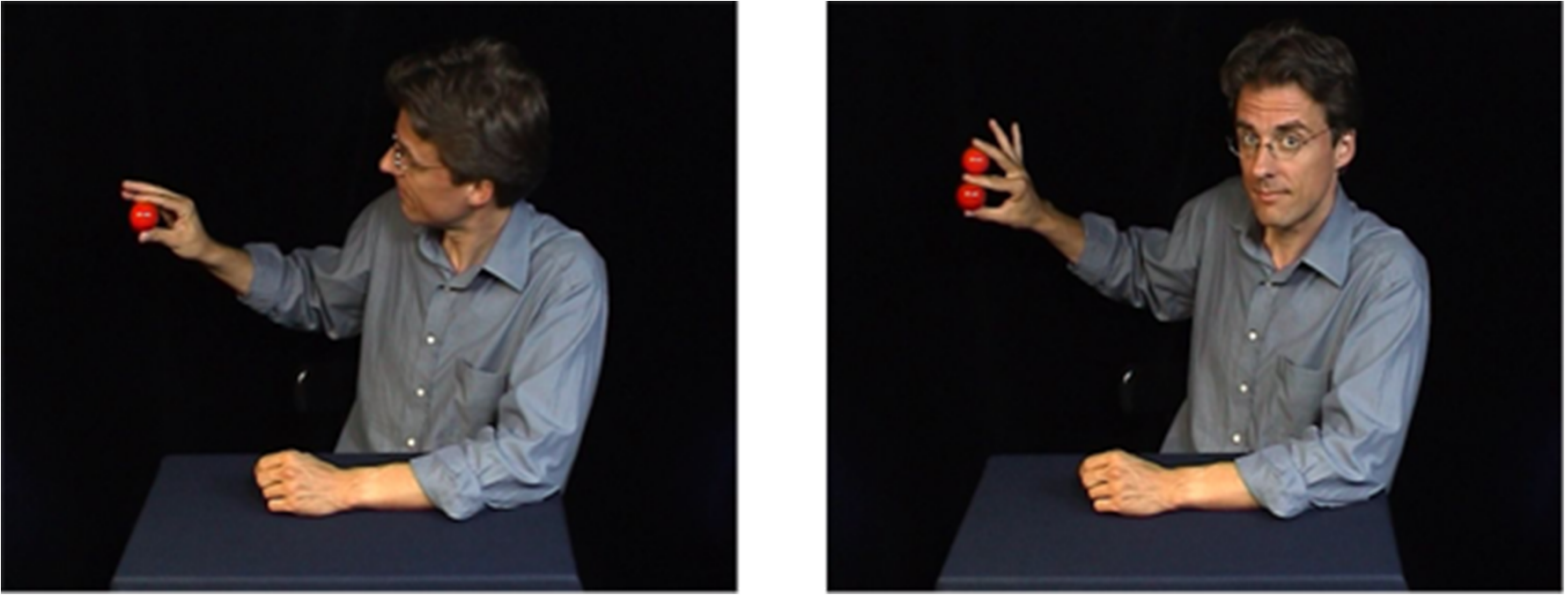
Example trick from the stimulus set. Screenshots from the initial (left) and the final (right) phase of the trick. In the video clip, the magician lets the little red ball multiply by simply shaking his hand. (see also http://www.youtube.com/watch?v=3B6ZxNROuNw for another example video clip)

In the following description of the experiment, we will give an example of one magic trick from our stimulus set (the “Ball Trick”). In this trick, the magician shows a little red ball to the audience (see Figure 1). Apart from the ball, his hand is empty. He then holds the ball up in the air, shakes his hand and produces a second ball. The ball has seemingly multiplied. How can this be? Typical viewers of this trick perceive the balls incorrectly, assuming that both are round, solid objects. Amodal volume completion [32],[33] is thought to be the perceptual mechanism behind the “Ball Trick”. Although only the front side of the ball can be seen, prior knowledge suggests that it looks identical from all sides. This constitutes a false mental problem representation. In reality, only half a ball exists, or rather just the shell of a ball, empty, with the second, slightly smaller ball stuck inside. During the shake, the two balls get separated and the second ball “appears” next to the first.

To correctly understand or “solve” a magic trick, insight and restructuring must take place: the mental representation of the solid ball must be discarded and substituted with the concept of half a ball covering another one. Many magic tricks exploit the fact that it is very hard to overcome these automatic assumptions [34].

#### 2.2.2. Cues

**Figure 2.**
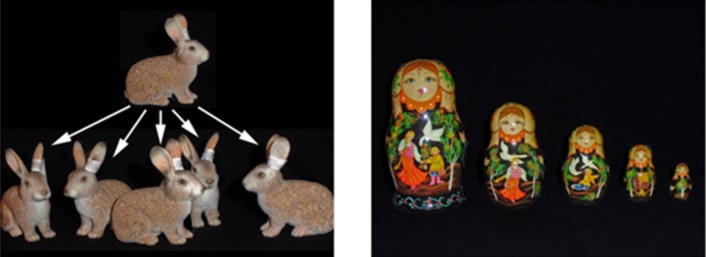
The two different cues used for the ball trick from Figure 1. Left: The congruent cue. The rabbits are multiplying like the red balls. Right: The incongruent cue. Matryoshka dolls are built so that the next size fits into the previous one, suggesting that multiple objects can be contained within one.

We have shown previously that solution rates, or the number of times an individual can correctly restructure their knowledge, can be increased by providing verbal hints after the viewing of the magic trick [29]. In a follow-up study, we found that pictorial cues were even more helpful than verbal cues [35]. In the present study, we therefore used only pictorial cues. In two pilot studies, we developed and refined the cues. In these pilot studies, magic tricks were presented one time and visual cues were given that either confirmed the perceptual mechanism behind the magic trick (congruent cue), or that could help them to find the real solution to the magic trick (incongruent cue—see below). Helpful, incongruent cues roughly doubled the solution rates as compared to unhelpful, congruent cues. In the present study, two different types of cues were implemented:

a) Congruent cue: This cue is congruent with the typical problem representation and does not offer any contradicting information. Basically, the principle of the magic effect that was just witnessed is repeated. For example, in the ball trick, the congruent cue (see Figure 2 left) illustrates the effect of “multiplying” by showing a number of little bunnies as the offspring of one bunny. Through this cue, participants are encouraged to stick with the initial problem representation of “several different solid balls” that prevents a correct solution. The cue does not trigger a cognitive conflict and is therefore not helpful.

b) Incongruent cue: This cue offers new information that contradicts participants' initial problem representation and thus leads to cognitive conflict—a prerequisite for restructuring the problem representation and gaining insight into the correct solution. It is therefore a helpful cue. For the ball trick, this is a picture of several Matryoshka dolls, inducing the idea of sticking objects into one another (Figure 2 right).

Only the incongruent cues will force participants to re-consider their perception of the problem, restructure it and thus enable them to gain insight into the trick.

#### 2.2.3. Trick selection

The set of 20 magic tricks (and their corresponding cues) for this study were selected from a larger set of 34 magic tricks based on the two pilot studies mentioned above. To be selected, the solving rate after the incongruent, helpful cue had to be ≥ 25%. Further, using the unrelated dataset from another study by Danek and Wiley [36] where participants (n = 70) had the possibility to see each trick up to three times before attempting to solve, we checked the spontaneous solution rates of the selected tricks after one single viewing. For the 20 selected tricks, these were all below 13% (with a mean of 2.9%).

### 2.3. Experimental design

The study was set up as a within-subjects design, so that each participant would be presented with 20 magic tricks (10 with an incongruent, helpful cue, 10 with a congruent, unhelpful cue). However, due to a numerical error in the seed randomization file, the actual ratio was 9:11 for each participant. Since conditions were switched after each participant, so that for the next participant, each trick would be shown in the alternate condition, this next participant had the ratio reversed (11:9). The next person had 9:11 again and so on. Therefore, across all participants, each trick was still presented in 50% of the cases with an incongruent cue, and in 50% with a congruent one. Trick order was randomized for each participant to control for learning effects.

Our data analysis was not impacted by this error, because each participant could solve an arbitrary number of tricks (individual solution rates ranged from 3 to 12 tricks). Therefore, the number of correct/incorrect solutions included in the analyses varied between participants. Further, we used mixed models, so that analyses were done on the level of observations and participants were modelled as a random effect. Overall, after excluding some more individual trials due to noise etc., the final distribution of trials across the two conditions was nearly equal (572 valid observations, 285 in the incongruent condition, 287 in the congruent condition, as stated in section 2.9.).

### 2.4. Procedure

**Figure 3.**
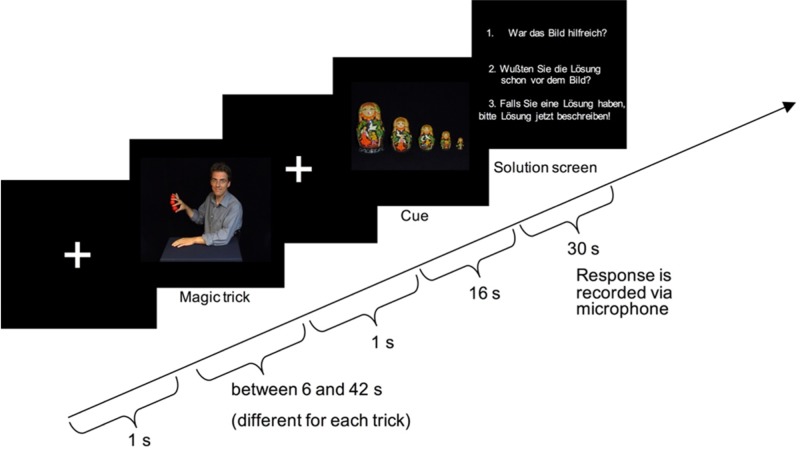
Sequence of one trial. Each trial started with a fixation cross followed by the magic trick of variable length (exposure phase). Then after an additional second of fixation, the cue phase started, where a cue was presented for 16 seconds, during which participants tried to solve the magic trick. Immediately after cue presentation, the solution phase began, where participants saw the three questions that they verbally responded to (see Procedure).

Each trial consisted of three phases, exposure, cue and solution phase, always in that order, in a block design (Figure 3). In the exposure phase, participants were presented with the problem once (one viewing of the magic trick clip), to set up a mental representation of the problem. This phase was between 6 and 42 seconds, depending on the clip. After a 1 second fixation, the 16 second cue phase followed, where the cue picture was presented. Finally, the solution phase immediately followed the cue phase, where participants verbally provided a solution and also answered two more questions within 30 seconds. All tasks were difficult problems with low solving rates, with the feature that the first, seemingly obvious problem representation does not lead to a correct solution. Thus, the problems used are typically not solvable after the first viewing.

In the scanner, participants were presented with 20 video clips of magic tricks, after an instruction to watch the clip carefully in order to find out how the trick works. They were also told that after each magic trick, a picture would be visible that could be either helpful for solving the magic trick or not. Either a helpful or unhelpful cue was then presented. Figure 3 shows the sequence of one trial. After each cue phase, the solution phase began. A solution screen appeared with the following three questions (translated from German): “1. Was the picture helpful? 2. Did you know the solution already before you saw the picture? 3. If you have a solution, please describe it now!” Participants were instructed to answer the questions verbally by speaking into a microphone. With insight problems, free verbalisation is the only way for participants to provide behavioral feedback that can be used for further analysis, which is why we chose this method. We separated the cue and the solution phases in order to temporally segregate the verbalisations and motor planning from the problem solving itself and to reduce motion confounds in the phase of the trial we were interested in. Together with the preparation phase (safety instructions for participant and practice trials) and the anatomical scan, each scanning session lasted about two hours.

### 2.5. Coding of solution accuracy

Using a coding manual (compiled with the help of the magician), participants' solutions were coded as correct (methods that the magician actually used or alternative methods verified as plausible) or incorrect (partial solutions, implausible methods, or impossible solutions with respect to the conditions seen in the video clip) by two independent raters. The two-way random intraclass correlation coefficient (absolute agreement), ICC (2, 2) was 0.97 with a 95% confidence interval of [0.97;0.98] indicating an excellent level of agreement according to the conventions set out in Koo and Li [37]. Conflicting cases were resolved by a third rater.

### 2.6. Experimental setup

The experiment, timing and synchronisation was programmed in python, presented on a Dell Latitude E6530 (64 bit) laptop computer running Windows 7. Magic clips and cues were presented to participants in the MRI machine via a back-projection system from a projector (Christie LX40) with True XGA 1024 × 768 resolution. While the solution screen was visible, participants could verbally respond to the three questions given. Their responses were recorded by an MRI compatible optical microphone (Sennheiser MO 2000, Wedemark, Germany) and amplifier (Sennheiser MO243 2000 CU) directly onto the same laptop computer. Only the 30 seconds of the solution phase were recorded.

### 2.7. Imaging data acquisition

Functional imaging data were acquired on a 3T MRI Scanner (Signa HDx, GE Healthcare, Milwaukee, WI, USA) with a standard 8-channel head coil. A BOLD-sensitive gradient echo echo-planar-imaging sequence was used to collect 597 volumes thirty-seven contiguous transverse slices of functional data (TR 2.0 s, TE 40 ms, flip angle 80 deg. Matrix 64 × 64 voxel, FOV 200 mm, 3.125 × 3.125 mm within-slice voxel size, 3.5 mm slice thickness, no gap). The first four functional images were not reconstructed to avoid T1 effects and are therefore not part of the final volume number. The experiment started synchronized to the fifth functional image. An additional 3D T1-weighted high-resolution structural image with 0.8 × 0.8 × 0.8 mm isotropic voxel size, was collected after functional imaging for normalization and visualization purposes.

### 2.8. Imaging data analysis

Functional imaging data were analyzed using SPM12, (version 7219, Wellcome Department of Imaging Neuroscience, University College London) on Matlab 8.2.0.701 (R2018a). The SPM12 default settings of each preprocessing step are used, unless otherwise specified. To improve coregistration performance, all images were manually reoriented so that the origin was set to the anterior commissure prior to preprocessing. Then the functional volumes were slice time corrected, realigned to the first volume of the first run and then to the mean across all runs. They were then coregistered to the anatomical image from each subject. The anatomical image was segmented into tissue probability maps based on standard stereotaxic space [Montreal Neurological Institute (MNI)], creating tissue subject-specific probability maps for grey matter, white matter, cerebrospinal fluid (CSF) bone and soft tissue. The inverse deformation field created during segmentation was used to normalize the functional volumes. Images were resampled to 2 × 2 × 2 mm isotropic voxels during normalization. Noise was reduced by smoothing the functional data using an 8-mm full-width at half-maximum Gaussian kernel.

Functional data were analyzed in each single subject block design using univariate multiple regression. The three experimental phases, the magic trick exposure, the cue phase and the solution phase, were all modeled as blocks with length: variable, 16 s, and 30 s respectively. However, we focused on the cue phase of the experiment (Figure 3), dividing this phase of the trials into separate regressors depending on the experimental condition, because the cue phase was where we expected both cognitive conflict and restructuring to occur in this design. The study was conceived as a 2 × 2 (congruence x solved) factorial design, which was originally carried through in the data analysis. For this first analysis, four regressors were created corresponding to trials that were congruent solved, congruent unsolved, incongruent solved and incongruent unsolved. Individual subject regression models included these four regressors, as well as the regressors of no interest in the final regression models used below. When analyzing the subject reports however, we found a higher rate of solving the tricks prior to the cue than in the pilot studies. We wanted to remove the previously solved trials for the question of restructuring. However, with the 2 × 2 factorial design it would have resulted in regressors in many subjects with 0–2 trials. We therefore chose instead to analyze the data as two separate models, one to test for brain areas corresponding to cognitive conflict, and one to test for regions that are recruited during restructuring.

In the first model, we modeled congruent and incongruent cue trials as separate regressors of interest to examine the brain regions recruited during cognitive conflict. All trials were categorized by the cue, independent of how they were solved. In the second model, we modeled the cue phase separately for three different types of trials. One regressor corresponded to trials in which the participant solved the magic trick correctly, a second for trials in which the participant did not solve the trick or solved it incorrectly, and one for trials in which the trick was solved before the cue phase. Both models additionally included movement parameters that modeled residual BOLD signal variability. Data were high-pass filtered (cutoff frequency = 0.0078 Hz) to minimize slow scanner related drifts. Global changes were removed by proportional scaling. For each subject, we computed subtractive contrasts of interest (Model 1: incongruent–congruent, Model 2: correctly solved–incorrectly solved) which were then entered into two group-level general linear models. T-tests for the difference between incongruent and congruent, and correct and incorrect were used to test for significant activity at the group level. Because of inter-subject differences in the solving rates, solving rate was additionally added as a regressor to the correct-incorrect group model.

The significance level was set to *p* < 0.05, FWE corrected at the cluster level. First, a voxel-level primary threshold of *p* < 0.001 was used to create clusters. Then under the null hypothesis of no signal, random field theory was used to estimate the largest null cluster size, given the smoothness of the data [38],[39]. Only voxels within the brain mask and outside of a CSF-mask (created by thresholding the MNI-template CSF probability map at 0.5) were considered in the analysis.

Anatomical regions were identified using the Anatomy toolbox (Version 2.2b) [40] for SPM and cross-checked with activity patterns in Neurosynth (http://neurosynth.org/). For frontal and prefrontal regions that have alternative labeling schemes than in the Anatomy toolbox, such as the dorsolateral prefrontal cortex (DLPFC), we compared our results to the MarsAtlas [41], the Brannetome Atlas (http://atlas.brainnetome.org/index.html) and to the delineations in the literature [42],[43].

### 2.9. Behavioral data analysis

For all analyses of behavioral data, mixed effects models were used. This approach has the advantage that it takes into account the hierarchical structure of the present data, in this case by modelling participants as random effects, fitting random intercepts for participants.

In total, 30 participants were presented with 20 tricks which yielded 600 observations. On the level of individual observations, seven observations could not be used for the analysis, because the low audio quality of the recording made it impossible to determine participants' responses. For the behavioral analysis, an additional 21 observations were excluded because participants indicated that they were already familiar with the solution to the trick, leaving 572 valid observations (285 in the incongruent condition, 287 in the congruent condition). Of those, 29.0% (166 observations) were not solved (i.e. no answer provided), 42.3% (242 observations) were correctly solved, and 28.7% (164) were incorrectly solved.

## 3. Results

### 3.1. Manipulation check

We first checked whether participants in the two cue conditions differed in their perception of the cue by using a mixed-effects model to perform a binary logistic regression on the question of how helpful the picture was (a categorical measure). We included cue condition as fixed effect and fitted random intercepts for subjects (*Z* = 1.30, *p* = 0.20). As intended, the helpful cue was perceived as more helpful (*M* = 0.72, *SD* = 0.45) than the unhelpful cue (*M* = 0.26, *SD* = 0.44), with *F*(1, 570) = 107.33, *p* < 0.001, Cohen's *d* = 0.87 (level of analysis is observations, number of observations = 572).

### 3.2. Behavioral results

An analogous analysis was run on the variable solution correctness. As expected, helpful, incongruent cues led to higher solution rates (*M* = 0.59, *SD* = 0.49) than the unhelpful, congruent cues (*M* = 0.26, *SD* = 0.44), with *F*(1, 570) = 58.81, *p* < 0.001, Cohen's *d* = 0.64 (number of observations = 572).

### 3.3. fMRI analyses

#### 3.3.1. Cognitive conflict

**Figure 4.**
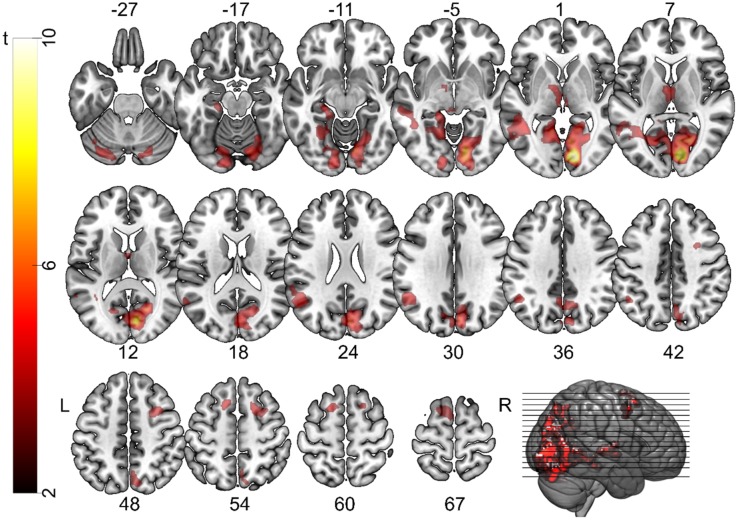
Activity patterns for cognitive conflict. Areas significantly more active during presentation of the incongruent cues compared to the congruent cues. Activity is thresholded at *p* < 0.001 voxel-wise and *p* < 0.05 FWE-corrected cluster-wise significance levels and overlaid onto a brain extracted version of the MNI152 template brain.

We first examined the influence of cue on brain activity by comparing the activity during incongruent cues and congruent cues. We found no significant brain activity for congruent cues compared to incongruent cues. This is in line with the idea that the congruent cues do not provide any additional knowledge to the participant; they represent the most common perceptual model of the trick. Incongruent cues, on the other hand, provide a conflict to the perceptual model of the magic trick. Indeed, during these trials, we found significantly higher brain activity predominantly in posterior brain areas, bilaterally, extending into the thalamus, hippocampus and temporal lobes (Figure 4). In addition, bilateral frontal activity could be seen, including the frontal eye fields [44] and extending into the dorsolateral prefrontal cortex (DLPFC). A list of the clusters of activity can be found in Table 1. Most of the activity was found in early visual areas. This is surprising, given that, although the same cues were used for each magic trick, each participant had a different randomization of what magic tricks were paired with congruent or incongruent cues. This means that although each trick had only one congruent and one incongruent cue, different participants saw different parings. Therefore, the effects we see are not likely related to visual differences in the cues themselves.

**Table 1.** Brain regions recruited during cognitive conflict. Peak voxels and corresponding brain regions that were significantly more active for incongruent vs. congruent cues. Magic tricks that were familiar to the participant were removed from the analysis. IPL: inferior parietal lobule, hOc1-3: human occipital cytoarchitectonic areas 1–3, corresponding to V1, V2 and V3 respectively, PGa, PFm & PFcm, cytoarchitectonically distinct areas of the inferior parietal lobe [45]. Coordinates are given in mm MNI space. Secondary peaks that do not have cluster sizes or p-values are sub-peaks within the current cluster.

Anatomical region		Cluster size	Coordinates (MNI) (mm)			Peak voxel		p-value (clus.)
	
		# voxels	x	y	z	T-value	z-score	p(FWE-corr)
R lingual gyrus, visual cortex, V1, BA17, Area hOc1 (V1)		7668	14	-82	4	9.86	6.48	0
	R Area hOc2 (V2), Area hOc3 (V3)		10	-64	4	6.6	5.12	
	R Area hOc1 (V1)		22	-66	10	6.59	5.11	
	R lingual gyrus, anterior -towards medial temporal lobe		14	-54	4	6.19		
	R lingual gyrus, anterior - towards medial temporal lobe		16	-52	2	6.09		
	R precuneus		6	-82	26	6.03		
L Area hOc3v [V3v]		924	-12	-88	-6	5.27	4.38	0
	L cerebellum, lobule VIIa, Crus I & hOc3 (V3)		-14	-90	-14	5.23	4.35	
	L cerebellum, lobule VIIa, Crus I		-28	-80	-26	4.98	4.2	
	L cerebellum, lobule VI		-16	-76	-16	4.86		
	L cerebellum, crus I		-38	-66	-30	4.03		
	L cerebellum, crus I		-40	-64	-24	3.91		
L middle temporal gyrus, Area PGa (IPL)		517	-48	-56	26	5.24	4.36	0.003
	L angular gyrus, Area PF (IPL) into Area PFm (IPL)		-48	-54	38	4.76	4.06	
	L superior temporal gyrus, and into Area PFcm (IPL)		-60	-42	24	3.7	3.32	
	L superior frontal gyrus, Area 6, dorsolateral	294	-16	12	60	5.18	4.32	0.032
	L superior frontal gyrus		-14	14	56	5.03		
	L posterior-medial frontal gyrus, dorsomedial prefrontal cortex (dmPFC)		-6	6	68	4.02	3.55	
	L superior frontal gyrus		-16	6	68	3.83	3.41	
R superior frontal gyrus		341	20	8	54	4.76	4.06	0.019
	R superior frontal gyrus, Area 6		18	16	58	4.7	4.02	
	R middle frontal gyrus, dorsolateral prefrontal cortex (DLPFC)		32	8	48	4.48	3.87	

#### 3.3.2. Restructuring (correct vs. incorrect)

**Figure 5.**
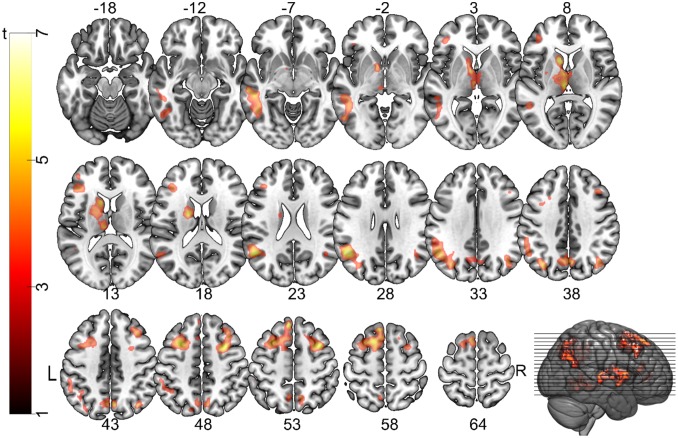
Activity patterns for restructuring. Areas significantly more active during the cue phase on correct trials compared to incorrect or not solved trials. Brain activity is more widespread and less posterior than in the previous contrast. Activity is thresholded at *p* < 0.001 voxel-wise and *p* < 0.05 FWE-corrected cluster-wise significance levels and overlaid onto a brain extracted version of the MNI152 template brain.

In a second analysis step, we compared the brain activity on trials where participants correctly solved the magic trick to trials where the magic trick was not correctly solved (either not solved or incorrectly). Trials in which participants reported to have solved the trick before the cue period started (see the solution phase in Figure 3) were modelled separately as trials of no interest, but not included in the comparison between correct and incorrect trials. No brain regions were significantly more active during incorrect trials compared to correct trials. In contrast, a widespread network of brain regions across the entire brain were recruited for correct trials compared to incorrect trials. Frontal, temporal and parietal cortical regions were significantly active, as well as the thalamus and the basal ganglia (Table 2, Figure 5).

The brain regions partially overlapped with regions that were significantly more active during incongruent cues. These regions include the frontal eye fields extending into the DLPFC and the medial temporal gyrus bilaterally. The early visual areas were no longer active for restructuring, but rather higher visual and parietal areas. Many more frontal regions were active during restructuring than during cognitive conflict.

**Table 2.** Brain regions active during restructuring. Peak voxels and corresponding brain regions that were significantly more active on correct trials than on incorrect trials. Trials that were solved before the cue was presented were not included in this contrast. IPS: inferior parietal sulcus, FG: frontal gyrus, MFG: medial frontal gyrus, SPL: superior parietal lobe, IFG: inferior frontal gyrus, MTG: medial temporal gyrus, ITG: inferior temporal gyrus. Coordinates are given in mm MNI space. Secondary peaks that do not have cluster sizes or p-values are sub-peaks within the current cluster.

Anatomical region		Cluster size	Coordinates (MNI) (mm)			Peak voxel		p-value (clus.)
		# voxels	x	y	z	T	Z-score	p(FWE-corr)
L caudate nucleus		1214	-10	14	10	6.86	5.21	0
	L thalamus, temporal and prefrontal part		-2	-20	6	6.19	4.87	
	L putamen		-12	2	-4	6.04	4.79	
	L middle temporal gyrus, Area PGa (IPL)	1349	-48	-56	28	6.41	4.99	0
	L middle temporal gyrus		-52	-56	24	6.34		
	L middle occipital gyrus, possibly Area PGa (IPL)		-34	-72	36	5.04	4.22	
	L inferior parietal lobule, Area hIP1 (IPS)		-38	-52	46	4.31	3.74	
	L supramarginal gyrus, Area PF (IPL)		-56	-44	36	4.30		
	L supramarginal gyrus, Area PFm (IPL)		-54	-52	40	4.22		
L superior medial gyrus, rostral medial prefrontal cortex (PFrm), Area 9m		1440	-6	36	54	6.39	4.98	0
	L superior frontal gyrus, dorsomedial prefrontal cortex (dmPF), Area 8m		-14	14	58	5.81	4.67	
	L middle frontal gyrus, dorsolateral prefrontal cortex (DLPFC)		-30	12	48	5.69	4.6	
	L posterior frontal gyrus, dorsomedial prefrontal cortex (dmPFC)		-8	18	62	4.75		
	L posterior medial gyrus, dorsomedial prefrontal cortex (dmPFC)		-12	6	56	4.71		
	L posterior medial gyrus, dorsomedial prefrontal cortex (dmPFC)		-4	14	64	4.58		
R middle frontal gyrus, dorsolateral prefrontal cortex (DLPFC)		661	36	8	52	5.95	4.75	0
	R middle frontal gyrus, dorsolateral prefrontal cortex (DLPFC)		30	6	48	5.8		
	R middle frontal gyrus, dorsolateral prefrontal cortex (DLPFC)		38	26	42	4.6	3.94	
	R middle frontal gyrus, dorsolateral prefrontal cortex (DLPFC)		36	20	48	4.43	3.82	
	R middle frontal gyrus, dorsolateral prefrontal cortex (DLPFC)		32	30	46	4.39		
	R superior frontal gyrus, dorsolateral prefrontal cortex (DLPFC)		18	20	54	4.21		
R precuneus		602	4	-70	42	5.67	4.59	0
	R precuneus		10	-60	52	4.35	3.77	
	L precuneus, possibly Area 7P (SPL)		-6	-62	54	4.12	3.61	
	L superior parietal lobule		-14	-78	48	3.82		
Cerebellar vermis & lobule V		384	2	-68	2	5.33	4.39	0.006
L Middle temporal gyrus		859	-52	-34	-8	5.23	4.33	0
	L middle temporal gyrus		-60	-28	-6	5.23	4.33	
	L inferior temporal gyrus, possibly Area FG4		-46	-48	-4	4.97	4.17	
	L inferior temporal gyrus		-48	-54	-6	4.82		
	L inferior temporal gyrus, FG4		-46	-56	-8	4.69		
	L inferior temporal gyrus		-54	-66	0	4.57		
L anterior insular lobe		316	-30	20	4	4.94	4.15	0.013
	L inferior frontal gyrus (pars Orbitalis)		-26	24	-4	4.76	4.04	
	L inferior frontal gyrus (pars Orbitalis)		-32	22	-8	4.25		
	L temporal pole		-52	16	-6	4.21	3.67	
	R angular gyrus, Area PGp (IPL)	266	38	-72	42	4.86	4.1	0.026
	R angular gyrus, parts of Area PGp (IPL)		42	-68	36	4.58	3.93	
	R middle temporal gyrus		42	-56	26	3.72	3.32	
L inferior frontal gyrus (pars Triangularis), ventrolateral prefrontal cortex (VLPFC)		356	-38	32	20	4.45	3.84	0.008
	L inferior frontal gyrus (pars Triangularis), ventrolateral prefrontal cortex (VLPFC)		-40	30	14	4.38		
	L middle frontal gyrus, ventrolateral prefrontal cortex (VLPFV)		-42	48	12	4.31	3.74	
								

## 4. Discussion

In this study, we systematically examined individual components of insight problem solving by introducing two types of cues that “prime” the participants to either maintain their perceptual belief (congruent cue) or to change their perceptual belief (incongruent cue) for the mechanism behind various magic tricks. In two pilot studies, these cues were developed and refined and magic tricks were chosen for their low spontaneous solution rates without cues and their intermediate solving rates with incongruent cues. Then in a separate experiment we tested naïve participants on these tricks and cues and measured their brain activity with fMRI. Behaviorally, across all participants, just under half of all of the trials were correctly solved. The incongruent cues were reported to be more helpful and had higher solution rates than the congruent cues. This finding supports the hypothesis that a cue which provides information which is incongruent with the solvers' initial mental representation of the problem triggers cognitive conflict and a subsequent restructuring of the problem representation which then often leads to a correct solution. Similarly, we found no significant brain activity for congruent or incorrect trials, compared to incongruent or correct trials, respectively. We did however find a predominantly visual network of activity for cognitive conflict (as reflected by an increased activity for incongruent trials), reflecting an increase in attentional control in the conflict situation on a perceptual level. Finally, a widespread frontal, parietal and subcortical network was active during restructuring, as reflected by an increased activity for correctly solved magic tricks.

Behaviorally speaking we can confirm and build on previous work that magic tricks provide a structured method for examining insight [1],[29]. Magic tricks have been refined over centuries to reliably produce a state of incomprehension (and awe) after the first observation of a trick. Magicians skillfully lead observers towards an incorrect mental representation of what is happening which does not allow them to see through the magic trick. Here we can say that with the incongruent cues, an almost 50% solving rate could be achieved, which makes comparisons between correct and incorrect solutions, also for neuroimaging or electrophysiological experiments, more feasible than many other insight problem solving tasks. The increase in solving rates was due to the introduction of an incongruent cue that induced a cognitive conflict - a prerequisite for restructuring and, in this paradigm, for correct solutions. Our behavioral results are therefore in support of our neuroimaging design.

The congruent cues which were specifically designed to be unhelpful worked as intended and led to rather low solving rates. This can be discussed in the light of other recent findings from the magic trick domain: Thomas et al. [46],[47] demonstrated that exposure to a false solution prevented participants from discovering the true solution to a magic trick. This effect persisted even after they had been informed about the incorrectness of that false solution. It is possible that the congruent cues may have acted in a similar manner: Although they are not false solutions, the congruent cues strengthen the initial, inappropriate view of the magic trick and make it more difficult for problem solvers to break away from this view.

### 4.1. Neural correlates of cognitive conflict

We defined cognitive conflict as the brain activity during incongruent cue types compared to congruent cue types, as the congruent information contradicts participants' initial problem representation. We found a predominantly posterior visual network of brain regions, including the left hippocampus, regions of the temporal cortex, the thalamus and the frontal eye-fields bilaterally together with the DLPFC (Figure 4). These areas together suggest that the cognitive conflict aspect of insight problem solving primarily involves perceptual conflict. These regions have been found in activities such as visually guided behavior [48], control of spatial attention [49], perspective taking [50] and many other aspects of visual cognition [51]—although see the Limitations section for possible confounds with our design. Interestingly, the early visual areas were more active during incongruent cues, although the cues themselves were not different in their visual properties, and even differed between participants, depending on what magic trick was associated with a congruent or incongruent cue.

If we consider the brain regions that were active for cognitive conflict in a network setting, two predominant networks are active, the visual network, together with self-referential components of the default mode network including the temporal cortical areas and the angular gyrus [52]. Large portions of the default mode network have been shown to be active comparing solvable to unsolvable problems in the Compound Remote Associates Task in a recent study by Kizilirmak et al. [53] which can be compared to our cognitive conflict contrast. Contrary to Kizilirmak et al., the solution is not presented at this point, and therefore mind-wandering as in resting-state fMRI is not likely to be the cause of the DMN activity. There are two possible explanations for the activity in the default mode network during insight problem solving. Insight events benefit from removing constraints [10], or cessation of attempts to solve the problem, in which the mind must relax allowing for spontaneous restructuring and potential solution events. Alternatively, the DMN activation pattern we find may support associative memory. The posterior part of the DMN has also been termed the parietal memory network (PMN) [54] for its role in memory processes.

This explanation for the DMN activity complements the visual activation pattern found. In our cognitive conflict condition, the conflict arises from the initial problem representation and the incongruent cue. The participants would need to remember the magic trick that was given and associate that trick with the new information from the incongruent cue. This produces a perceptual conflict [55], in the Bayesian sense that prior sensory information, or visual memory, is in conflict with the current sensory information and the visual system attempts to resolve this conflict and reinterpret the sensory information that is received. Early as well as higher visual areas are thought to be involved in both associative visual memory and perceptual conflict resolution [56].

There are a number of important differences between cognitive conflict as it has been used in the literature, and the cognitive conflict that arises from the incongruent cues in magic insight problems. For instance, the flanker task is a classical example of cognitive conflict, where distractor words with the opposite semantic meaning flank the target word [57]. This conflict involves active control of cognition towards the target word and away from the incongruent flanker words, actively suppressing them, whereas our cognitive conflict contrast involves relinquishing control, allowing for new associations to be made. We believe this explains why we do not see more frontal activity in this contrast (although the DLPFC, a typical cognitive control region, was active), in particular in the anterior cingulate cortex (ACC) and the ventrolateral prefrontal cortex (VLPFC) as was found previously [57]. If the ACC is responsible for conflict monitoring and decision making [58], these processes do not necessarily occur in our cognitive conflict contrast.

In a meta-analysis on insight, Sprugnoli et al. found activity in temporo-occipital regions, in the middle temporal gyrus and in the frontal eye fields [25] that is specific to insight. The left angular gyrus (AG) has been found in insight problem solving in a comparable way. Kizilirmak et al. [59] contrasted correct solution words with pseudo-solution words, independent of participants' responses, similar to our cognitive conflict contrast. Another study [60] contrasted solved technical problems with a related “heuristic prototype” with unsolved technical problems with an unrelated “heuristic prototype”. Both of these studies reported left angular gyrus activity.

A review by Seghier [61], based on meta-analyses and focusing on angular gyrus activity in healthy populations, describes the AG as a major connecting hub which is activated by a large number of different tasks. A key function which most consistently activates the left AG is semantic processing [62], also for visual stimuli [63]. In particular, the left AG is thought to be involved in concept retrieval and conceptual integration [62] which fits with the present task where a helpful concept (illustrated by the helpful, incongruent cue) needed to be retrieved and understood and then integrated into the mental representation of the magic trick. More interestingly, the AG was consistently found for conflict resolution [61]. In contrast to the right AG, the left AG is not activated by all conflicts (such as the classical go/no-go paradigm), but only by conflicts in a semantic context. Ye and Zhou [64] reported that left AG activity was triggered only by a conflict between plausible and implausible sentential representations.

The following possible function of the AG in processing incongruent vs. congruent cues in problem solving emerges: Multisensory input, in this case the perception of the pictorial cues, from lower brain levels such as the visual cortex, is integrated in the AG. Top-down predictions, based on prior knowledge, shape the integration. In the case of incongruent cues, the prediction (i.e. the mental problem representation) is at odds with the new information provided by the cue, causing cognitive conflict and a prediction error [65]. This does not happen for congruent cues since these simply reiterate the principle of the magic trick, which is in agreement with the viewer's existing problem representation and thus does not cause any conflict. This explains why left AG is activated only by the incongruent cues as compared to the congruent ones. It is important to note here as well, that we found no significant activity for congruent cues compared to incongruent cues, which is also in line with this theory.

### 4.2. Neural correlates of restructuring

To examine restructuring, we compared trials in which participants were able to arrive at the correct solution to trials in which they were not. Here, the activity was more widespread than for cognitive conflict, in areas including the basal ganglia, the insula, parietal and temporal regions as well as more frontal regions. In general, the regions found in the two analyses did not show a large amount of overlap which indicates that we were actually tackling two different components of the insight process with the chosen contrasts.

Restructuring is closer to the actual “Aha!” or insight moment than is cognitive conflict. Therefore, more of the regions found for restructuring were also found in the meta-analysis of insight by Sprugnoli [25]. The middle and inferior frontal gyri (IFG, MFG) were found and suggested to be associated with memory, inhibition and task switching [66]. The anterior insula was also active during restructuring, a region known for its involvement in interoception and self-awareness [67].

The left medial frontal gyrus as well as the left middle temporal gyrus have also been found in a previous study [68], based on a comparable, but not identical, contrast. Tian et al. [68] compared the successful guessing of Chinese logogriphs to unsuccessful guessing. In contrast to the present study, participants did not actually provide the solution, instead, after trying to solve, the correct answer was presented and participants indicated whether they had thought of this answer or not. The authors interpreted this activity as related to breaking mental set or restructuring. Moreover, the medial frontal gyrus has been shown to be involved in creativity in another meta-analysis [69].

The left middle temporal gyrus (MTG) which represented one of the largest clusters for the restructuring contrast had previously been found by a recent study by Tik et al. [17] using the same contrast (i.e. solved vs. unsolved trials). Further, the MTG was one of the largest clusters of the insight network as outlined by Sprugnoli et al. [25]. The MTG also belongs to the salience network which supports dynamic switching between the default mode network and the executive control network [70]. Further, Beaty et al. [71] have implicated the MTG in creative cognition which fits with the idea of achieving a completely new view on a problem (restructuring it).

The activity found during restructuring partially overlapped with the regions found for cognitive conflict. This makes sense since we always considered the entire cue phase and did not look at restructuring only during or after it took place. The angular gyrus, middle temporal gyrus and frontal eye fields were present in both activity patterns, suggesting that activity in these regions are maintained throughout cognitive conflict and restructuring. All three regions were present in the meta-analysis of insight [25]. The dorsomedial thalamus was also active during cognitive conflict and restructuring but was not present in the meta-analysis. However, it is a region that is embedded in the salience network [72] and may therefore be relevant for both cognitive processes.

A number of regions also changed their statistical significance between the two cognitive processes. The early visual areas no longer reach significance for the correct trials, suggesting that visual attention, prediction error and comparing sensory input is no longer as relevant for restructuring as it was during cognitive conflict. Instead, frontal and parietal regions, together with the basal ganglia survived thresholding during restructuring. If we consider these regions from a network perspective, then the salience, executive function, and default mode network are all represented more during restructuring than during cognitive conflict. The medial prefrontal cortex and the precuneus are two highly connected hubs of the executive and default mode networks, respectively. Previous work shows that, although these two networks are anti-correlated at rest, a higher coupling between these two networks, and also with the anterior insula of the salience network supports the production of creative ideas [71]. It was suggested that the salience network helps to reallocate resources and allow for dynamic switching between the default mode network and the executive control network to promote creativity and insight [71],[25]. We can add that this happens already at the phase of restructuring but likely not during cognitive conflict.

Two previous studies have examined brain activity during magic tricks [30],[73]. Because both studies examined brain activity during passive viewing of magic instead of during the search for a solution, it is difficult to compare these studies to the current one. However, two regions of the brain are worth mentioning. First, both studies found the anterior cingulate cortex (ACC), a region that is often thought to be crucial for insight problem solving and many other cognitive tasks [67],[25]. This region was speculated to be specifically active for conflict detection [30],[73] which is likely why we do not find this region in our study, as the conflict detection had already occurred (since the cue directly pointed out the conflict). The basal ganglia, in particular the caudate nucleus, were also active during passive viewing of magic tricks and during restructuring. The caudate nucleus is thought to process changes in the contingency between an action and its outcome, primarily in order to perform successful goal-directed action [30],[74]. This fits with our current results as well, where we would suggest a change in contingency during restructuring but not during cognitive conflict.

### 4.3. Limitations

Although we designed this study to the best of our ability to address the questions of interest, there are a number of limitations we would like to mention. Most importantly, we chose not to try and determine the actual “Aha moment” or the moment in which the participants solved the magic trick. This was primarily to avoid the motor confound in the imaging data that we would have had, if we had allowed subjects to give a response at any time. It is likely that additional brain regions, including the ACC, may be active only for the time point of insight. For instance, the ACC was active for the individual time point of conflict detection in magic tricks [30],[73]. Only with this additional time point can we really understand the brain state at the moment of insight.

Based upon the present results, we believe that using eye-tracking to monitor gaze behavior during cue viewing would provide additional important information about the different components of insight problem solving. The frontal eye fields were active for cognitive conflict and restructuring, suggesting a difference in gaze behavior between congruent and incongruent trials as well as between correct and incorrect trials. Additionally, pupillometry has been shown to relate to reward anticipation and correlate with activity in the salience network [75]. In future studies, recording pupil dilations may provide a time point of insight without a conscious motor response from participants.

## 5. Conclusion

In contrast to most neuroscientific studies investigating insight problem solving in its entirety, or in the single moment of the “Aha!” experience, the present study breaks down this complex process into two theoretically derived components to identify the neural correlates for each of them. Through this new approach, we succeeded in separating out the neural substrates for cognitive conflict from those for restructuring. This is not only a theoretically valuable result, but it may also help to resolve some of the inconsistency found across studies, as reflected in two current meta-analyses [22],[25]. We found that brain activity during cognitive conflict reflected processes related to prediction error, attention to the relevant cue-specific sensory domain, and the default brain state. Restructuring on the other hand, was related to an interplay between the default mode and the executive control networks, that may be modulated by the salience network. The angular gyrus, middle temporal gyrus, frontal eye fields and DLPFC were all active in both cognitive conflict and restructuring, suggesting a more overarching role of these regions in the whole insight process. These results demonstrate the benefit of breaking down insight problem solving into its constituent processes to understand how the brain orchestrates such a complex cognitive task.

## References

[1] Danek AH, Vallée-Tourangeau F (2018). Magic tricks, sudden restructuring and the Aha! experience: A new model of non-monotonic problem solving. Insight: On the origins of new ideas.

[2] Danek AH, Wiley J, Öllinger M (2016). Solving classical insight problems without Aha! experience: 9 Dot, 8 Coin, and Matchstick Arithmetic Problems. J Probl Solving.

[3] Bowden EM (1997). The effect of reportable and unreportable hints on anagram solution and the Aha! experience. Conscious Cogn.

[4] Cushen PJ, Wiley J (2012). Cues to solution, restructuring patterns, and reports of insight in creative problem solving. Conscious Cogn.

[5] Stroop JR (1935). Studies of interference in serial verbal reactions. J Exp Psychol.

[6] Luo J, Knoblich G (2007). Studying insight problem solving with neuroscientific methods. Methods.

[7] Ohlsson S (1984). Restructuring revisited: II. An information processing theory of restructuring and insight. Scand J Psychol.

[8] Smith SM, Sternberg RJ, Davidson JE (1995). Getting into and out of mental ruts: A theory of fixation, incubation, and insight. The nature of insight.

[9] Wertheimer M, Wertheimer M (1925). Über Schlussprozesse im produktiven Denken. Drei Abhandlungen zur Gestalttheorie.

[10] Knoblich G, Ohlsson S, Haider H (1999). Constraint relaxation and chunk decomposition in insight problem solving. J Exp Psychol Learn Mem Cogn.

[11] Ohlsson S, Keane MT, Gilhooly KJ (1992). Information-processing explanations of insight and related phenomena. Advances in the psychology of thinking.

[12] Ohlsson S (2011). Deep learning: How the mind overrides experience.

[13] Sandkühler S, Bhattacharya J (2008). Deconstructing insight: EEG correlates of insightful problem solving. PLoS One.

[14] Smith RW, Kounios J (1996). Sudden insight: All-or-none processing revealed by speed-accuracy decomposition. J Exp Psychol Learn Mem Cogn.

[15] Metcalfe J (1986). Feeling of knowing in memory and problem solving. J Exp Psychol Learn Mem Cogn.

[16] Metcalfe J, Wiebe D (1987). Intuition in insight and noninsight problem solving. Mem Cognit.

[17] Tik M, Sladky R, Luft CDB (2018). Ultra-high-field fMRI insights on insight: Neural correlates of the Aha!-moment. Hum Brain Mapp.

[18] Jung-Beeman M, Bowden EM, Haberman J (2004). Neural activity when people solve verbal problems with insight. PLoS Biol.

[19] Starchenko MG, Bekhtereva NP, Pakhomov SV (2003). Study of the brain organization of creative thinking. Hum Physiol.

[20] Bechtereva NP, Korotkov AD, Pakhomov SV (2004). PET study of brain maintenance of verbal creative activity. Int J Psychophysiol.

[21] Luo J, Niki K, Phillips S (2004). Neural correlates of the Aha! reaction. NeuroReport.

[22] Dietrich A, Kanso R (2010). A review of EEG, ERP, and neuroimaging studies of creativity and insight. Psychol Bull.

[23] Bowden EM, Jung-Beeman M (2003). Aha! Insight experience correlates with solution activation in the right hemisphere. Psychon Bull Rev.

[24] Kounios J, Beeman M (2014). The cognitive neuroscience of insight. Annu Rev Psychol.

[25] Sprugnoli G, Rossi S, Emmendorfer A (2017). Neural correlates of Eureka moment. Intelligence.

[26] Luo J, Niki K, Phillips S (2004). The function of the anterior cingulate cortex (ACC) in the insightful solving of puzzles: The ACC is activated less when the structure of the puzzle is known. J Psychol Chin Soc.

[27] Zhao Q, Zhou Z, Xu H (2013). Dynamic neural network of insight: a functional magnetic resonance imaging study on solving chinese ‘chengyu’ riddles. PloS One.

[28] Aziz-Zadeh L, Kaplan JT, Iacoboni M (2009). “Aha!”: The neural correlates of verbal insight solutions. Hum Brain Mapp.

[29] Danek AH, Fraps T, von Müller A (2014). Working wonders? Investigating insight with magic tricks. Cognition.

[30] Danek AH, Öllinger M, Fraps T (2015). An fMRI investigation of expectation violation in magic tricks. Front Psychol.

[31] Danek AH, Fraps T, von Müller A (2013). Aha! experiences leave a mark: facilitated recall of insight solutions. Psychol Res.

[32] Ekroll V, Sayim B, Wagemans J (2013). Against better knowledge: The magical force of amodal volume completion. Iperception.

[33] Ekroll V, Sayim B, Wagemans J (2017). The other side of magic: the psychology of perceiving hidden things. Perspect Psychol Sci.

[34] Ekroll V, Sayim B, Van der Hallen R (2016). Illusory visual completion of an object's invisible backside can make your finger feel shorter. Curr Biol.

[35] Pétervári J, Danek AH (under review). Problem solving of magic tricks: Guiding to and through an impasse with solution cues.

[36] Danek AH, Wiley J (2017). What about false insights? Deconstructing the Aha! experience along its multiple dimensions for correct and incorrect solutions separately. Front Psychol.

[37] Koo TK, Li MY (2016). A guideline of selecting and reporting intraclass correlation coefficients for reliability research. J Chiropr Med.

[38] Nichols TE (2012). Multiple testing corrections, nonparametric methods, and random field theory. NeuroImage.

[39] Woo C-W, Krishnan A, Wager TD (2014). Cluster-extent based thresholding in fMRI analyses: Pitfalls and recommendations. NeuroImage.

[40] Eickhoff SB, Stephan KE, Mohlberg H (2005). A new SPM toolbox for combining probabilistic cytoarchitectonic maps and functional imaging data. NeuroImage.

[41] Auzias G, Coulon O, Brovelli A (2016). MarsAtlas: A cortical parcellation atlas for functional mapping. Hum Brain Mapp.

[42] Carlén M (2017). What constitutes the prefrontal cortex?. Science.

[43] Nee DE, Brown JW, Askren MK (2013). A meta-analysis of executive components of working memory. Cereb Cortex.

[44] Lobel E, Kahane P, Leonards U (2001). Localization of human frontal eye fields: anatomical and functional findings of functional magnetic resonance imaging and intracerebral electrical stimulation. J Neurosurg.

[45] Caspers S, Eickhoff SB, Geyer S (2008). The human inferior parietal lobule in stereotaxic space. Brain Struct Funct.

[46] Thomas C, Didierjean A (2016). Magicians fix your mind: How unlikely solutions block obvious ones. Cognition.

[47] Thomas C, Didierjean A, Kuhn G (2018). It is magic! How impossible solutions prevent the discovery of obvious ones?. Q J Exp Psychol.

[48] Wright RD, Lawrence MW (2008). Orienting of attention.

[49] Moore T, Fallah M (2004). Microstimulation of the frontal eye field and its effects on covert spatial attention. J Neurophysiol.

[50] Wang H, Callaghan E, Gooding-Williams G (2016). Rhythm makes the world go round: An MEG-TMS study on the role of right TPJ theta oscillations in embodied perspective taking. Cortex.

[51] Vernet M, Quentin R, Chanes L (2014). Frontal eye field, where art thou? Anatomy, function, and non-invasive manipulation of frontal regions involved in eye movements and associated cognitive operations. Front Integr Neurosci.

[52] Greicius MD, Krasnow B, Reiss AL (2003). Functional connectivity in the resting brain: A network analysis of the default mode hypothesis. Proc Natl Acad Sci.

[53] Kizilirmak JM, Schott BH, Thuerich H (2019). Learning of novel semantic relationships via sudden comprehension is associated with a hippocampus-independent network. Conscious Cogn.

[54] Gilmore AW, Nelson SM, McDermott KB (2015). A parietal memory network revealed by multiple MRI methods. Trends Cogn Sci.

[55] Zacks JM, Speer NK, Swallow KM (2007). Event perception: A mind-brain perspective. Psychol Bull.

[56] Albright TD (2012). On the perception of probable things: neural substrates of associative memory, imagery, and perception. Neuron.

[57] Ochsner KN, Hughes B, Robertson ER (2009). Neural systems supporting the control of affective and cognitive conflicts. J Cogn Neurosci.

[58] Botvinick MM (2007). Conflict monitoring and decision making: Reconciling two perspectives on anterior cingulate function. Cogn Affect Behav Neurosci.

[59] Kizilirmak JM, Thuerich H, Folta-Schoofs K (2016). Neural correlates of learning from induced insight: a case for reward-based episodic encoding. Front Psychol.

[60] Dandan T, Haixue Z, Wenfu L (2013). Brain activity in using heuristic prototype to solve insightful problems. Behav Brain Res.

[61] Seghier ML (2013). The Angular Gyrus: Multiple Functions and Multiple Subdivisions. The Neuroscientist.

[62] Binder JR, Desai RH, Graves WW (2009). Where is the semantic system? A critical review and meta-analysis of 120 functional neuroimaging studies. Cereb Cortex.

[63] Vandenberghe R, Price C, Wise R (1996). Functional anatomy of a common semantic system for words and pictures. Nature.

[64] Ye Z, Zhou X (2009). Conflict control during sentence comprehension: fMRI evidence. Neuroimage.

[65] Spratling MW (2016). Predictive coding as a model of cognition. Cogn Process.

[66] Benn Y, Webb TL, Chang BPI (2014). The neural basis of monitoring goal progress. Front Hum Neurosci.

[67] Craig AD (2009). How do you feel—now? The anterior insula and human awareness. Nat Rev Neurosci.

[68] Tian F, Tu S, Qiu J (2011). Neural correlates of mental preparation for successful insight problem solving. Behav Brain Res.

[69] Boccia M, Piccardi L, Palermo L (2015). Where do bright ideas occur in our brain? Meta-analytic evidence from neuroimaging studies of domain-specific creativity. Front Psychol.

[70] Sridharan D, Levitin DJ, Menon V (2008). A critical role for the right fronto-insular cortex in switching between central-executive and default-mode networks. Proc Natl Acad Sci.

[71] Beaty RE, Benedek M, Barry Kaufman S (2015). Default and executive network coupling supports creative idea production. Sci Rep.

[72] Menon V (2015). Salience Network. Brain Mapp Encycl Ref.

[73] Parris BA, Kuhn G, Mizon GA (2009). Imaging the impossible: An fMRI study of impossible causal relationships in magic tricks. NeuroImage.

[74] Grahn JA, Parkinson JA, Owen AM (2008). The cognitive functions of the caudate nucleus. Prog Neurobiol.

[75] Schneider M, Leuchs L, Czisch M (2018). Disentangling reward anticipation with simultaneous pupillometry/fMRI. Neuroimage.

